# Tackling the glial scar in spinal cord regeneration: new discoveries and future directions

**DOI:** 10.3389/fncel.2023.1180825

**Published:** 2023-05-24

**Authors:** Areez Shafqat, Ibrahem Albalkhi, Hamzah M. Magableh, Tariq Saleh, Khaled Alkattan, Ahmed Yaqinuddin

**Affiliations:** College of Medicine, Alfaisal University, Riyadh, Saudi Arabia

**Keywords:** spinal cord injury, axonal regeneration, glial scar, neuroinflammation, astrocyte heterogeneity, microglia heterogeneity, fibroblast heterogeneity, neuroprotection

## Abstract

Axonal regeneration and functional recovery are poor after spinal cord injury (SCI), typified by the formation of an injury scar. While this scar was traditionally believed to be primarily responsible for axonal regeneration failure, current knowledge takes a more holistic approach that considers the intrinsic growth capacity of axons. Targeting the SCI scar has also not reproducibly yielded nearly the same efficacy in animal models compared to these neuron-directed approaches. These results suggest that the major reason behind central nervous system (CNS) regeneration failure is not the injury scar but a failure to stimulate axon growth adequately. These findings raise questions about whether targeting neuroinflammation and glial scarring still constitute viable translational avenues. We provide a comprehensive review of the dual role of neuroinflammation and scarring after SCI and how future research can produce therapeutic strategies targeting the hurdles to axonal regeneration posed by these processes without compromising neuroprotection.

## 1. Background

In response to tissue injury, the body swiftly seeks to restore homeostasis by minimizing damage spread and recovering normal tissue function. The wound healing process involves conserved and coordinated phases of hemostasis, inflammation, and remodeling. However, in the adult mammalian central nervous system (CNS), this healing process is prolonged and culminates in the formation of an injury scar characterized by a fibrotic core surrounded by a limitans border of astrocytes, termed the glial border or glial scar, in the lesion’s immediate penumbra (Adams and Gallo, 2018).

Central nervous system regeneration is notoriously poor after traumatic spinal cord injury (SCI) (Bradbury and McMahon, 2006). The injury scar was once viewed as the primary obstacle to successful regeneration, leading to numerous attempts to inhibit its essential components (Silver and Miller, 2004). However, contemporary research has largely moved past this notion, adopting a more comprehensive approach considering neuron-intrinsic properties. Advances in neural stem cell (NSC) transplantation and the administration of neurotrophic factors have achieved unprecedented levels of neural regeneration and functional recovery (Lu et al., 2012; Anderson et al., 2018), even progressing to early-phase clinical trials (Liu et al., 2022).

Conversely, strategies targeting scar components have not reproducibly yielded noteworthy beneficial effects in animal models (Zheng and Tuszynski, 2023). Moreover, genetic manipulations that deplete or attenuate glial or stromal cells in the glial scar have revealed numerous protective functions in SCI (Wahane and Sofroniew, 2022). There is also currently no FDA-approved drug targeting scar-associated neuroinflammation in the management of SCI. These observations prompt a critical question: is targeting the SCI scar beneficial, and should it remain a focus of future research? Answering this question requires a deeper understanding of the roles of various cells in SCI. This review discusses recent advancements in SCI cell biology, reflects on current study limitations, and proposes a trajectory for future research in this area.

## 2. Formation and composition of the glial scar

### 2.1. Primary and secondary spinal cord injury

Tissue response to injury begins with local vascular damage and the infiltration of blood-borne immune cells (Gurtner et al., 2008; Eming et al., 2014). The CNS injury response follows a similar pattern (Burda Joshua and Sofroniew Michael, 2014; Orr and Gensel, 2018; Bradbury and Burnside, 2019; Anjum et al., 2020; Hellenbrand et al., 2021).

**FIGURE 1:**
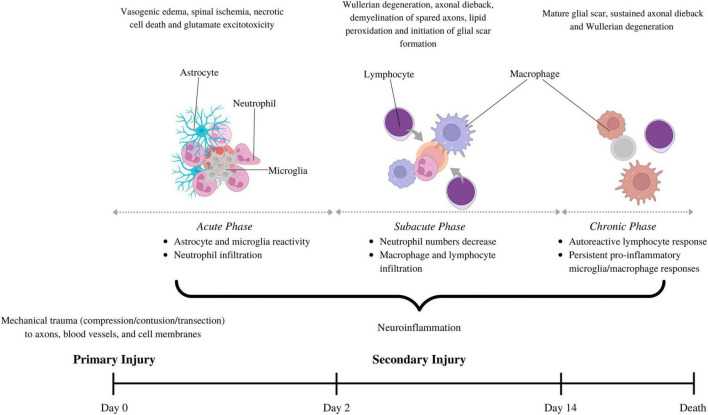
The pathophysiology behind spinal cord injury involves primary injury, describing initial mechanical trauma to the spinal trauma, and secondary injury, which sustains spinal cord damage. The secondary phase is further divided into acute (0–2 days), subacute (2–14 days) and chronic (>14 days), each with its own pathophysiological hallmarks. Neuroinflammation is a part of all the secondary injury phases, but the cell types involved vary. Astrocytes and microglia are the first to become reactive. They secrete cytokines/chemokines that recruit neutrophils in the acute phase. Neutrophil numbers subside in the subacute phase, coinciding with macrophage and lymphocyte infiltration. Macrophage and lymphocytes can stay elevated in the chronic phase to drive persistent inflammation and impair wound resolution. This figure was created with Biorender.com.

Spinal cord injuries are divided into primary and secondary injury mechanisms (Figure 1; Sekhon and Fehlings, 2001; Alizadeh et al., 2019). The primary injury can take many forms, all involving mechanical forces that disrupt several ascending and descending tracts, blood vessels, the blood-spinal cord barrier (BSCB), and cell membranes of neurons and glial cells (Tator and Fehlings, 1991; Tator, 1998; Rossignol et al., 2007). This leads to local tissue ischemia and necrotic cell death, manifesting as spinal and potential neurogenic shock, neurotransmitter and electrolyte imbalances, and the release of pro-inflammatory damage-associated molecular patterns (DAMPs) such as ATP and high-mobility group box-1 (HMGB1) (Bianchi, 2007; Tran et al., 2018b; Anjum et al., 2020).

These events give rise to a secondary injury response, a series of cellular, molecular, and biochemical mechanisms that chronically exacerbate tissue loss and impede functional recovery (Allen, 1911; Oyinbo, 2011; Fehlings et al., 2012). Traditionally, the secondary injury response to SCI has been categorized into acute [0–2 days-post injury (dpi)], subacute (2–14 dpi), and chronic phases (>14 dpi), each with unique and overlapping pathophysiological hallmarks. For example, the acute phase features vascular hemorrhage, vasogenic edema, necrotic cell death, neurotransmitter and electrolyte imbalance, and excitotoxicity (Oyinbo, 2011; Alizadeh et al., 2019). The subacute phase involves demyelination of spared axons due to continued oligodendrocyte apoptosis, Wallerian degeneration of the distal stump of transected axons, and axonal dieback of the proximal end. The chronic phase is typified by a mature SCI scar, comprising a fibrotic core—often containing a central cystic cavity—encircled by a glial scar of astrocytes and oligodendrocyte progenitor cells (OPCs). We refer readers to other reviews for additional information on the unique and overlapping disease processes occurring in these phases (Oyinbo, 2011; Alizadeh et al., 2019).

### 2.2. Neuroinflammation in spinal cord injury

Neuroinflammation refers to the induction of reactive states in various CNS cell types and the recruitment of circulating innate and adaptive immune cells (Bareyre and Schwab, 2003). Neuroinflammation is a salient feature of all the phases of secondary injury but varies with intensity, peaking in the acute and subacute phases (Oyinbo, 2011; Anwar et al., 2016). CNS resident cells, such as astrocytes and microglia, are the first to react to the primary injury site, secreting pro-inflammatory cytokines and chemokines that recruit blood-borne immune cells to the lesion epicenter and activate them (Fawcett and Asher, 1999; Schnell et al., 1999; Davalos et al., 2005; Rice et al., 2007; Burda and Sofroniew, 2014).

During the acute phase, neutrophils are recruited to the SCI lesion site, where they exert deleterious effects by producing reactive oxygen species (ROS), pro-inflammatory cytokines, and proteases, which exacerbate neuronal loss and neuroinflammation (Dinkel et al., 2004; Nguyen et al., 2007; Bi et al., 2021; Dolma and Kumar, 2021; Feng et al., 2021). However, some studies have reported that infiltrating neutrophils in SCI contribute to the resolution of neuroinflammation and create an environment conducive to axonal regeneration (de Castro et al., 2004; Stirling et al., 2009; Ghasemlou et al., 2010; Schreiber et al., 2013). A seminal paper by Stirling et al. demonstrated that depleting neutrophils in the acute phase of SCI worsens tissue damage, reduces local levels of growth factors such as vascular endothelial growth factor (VEGF) and fibroblast growth factor (FGF), and compromises functional recovery (David et al., 2009). Neutrophil numbers in the SCI lesion site begin to decline in the subacute phase, coinciding with the infiltration of monocyte-derived macrophages (MDMs) and adaptive B and T lymphocytes (Neirinckx et al., 2014).

Macrophages infiltrate the lesion site after 2–3 dpi, peaking around 7–10 dpi (Perry and Teeling, 2013; Andrew and Samuel, 2014). Macrophages originate either from circulating monocytes, termed MDMs, or CNS resident macrophages in the perivascular spaces and meninges. Reactive microglia and MDMs occupy distinct locations in the fibrotic scar, with MDMs at the center and reactive microglia in the periphery, interfacing with the astrocyte border (David and Kroner, 2011; Zhou et al., 2014; Wang et al., 2015). These cell types also differ temporally: microglia proliferate rapidly at the lesion site, peaking at 14 dpi, whereas MDMs peak at 7–10 dpi and again at 60 dpi (Popovich et al., 1997; Bellver-Landete et al., 2019; Milich et al., 2021). While the numbers of macrophages and microglia decline in the chronic phase, this resolution is incomplete, with phagocytic pro-inflammatory macrophages and reactive microglia persisting months after SCI onset and contributing to impaired wound healing (Fleming et al., 2006; Prüss et al., 2011).

Lymphocytes begin infiltrating the lesion site in the subacute phase and remain elevated in the chronic phase, driving autoimmunity and neuroinflammation (Jones, 2014; Allison and Ditor, 2015). At the lesion site, antigen-presenting cells such as macrophages present self-antigens to T-cells, thereby fostering a chronic autoimmune T-cell response (Jones, 2014). Autoreactive CD4+ T-cells can adopt a T helper-1 (Th1) type phenotype, secreting pro-inflammatory cytokines that induce pro-inflammatory/anti-repair microglia and macrophage polarization states (Yu and Fehlings, 2011). Autoreactive CD4+ T-cells can also stimulate humoral immune responses by promoting B-cell differentiation into plasma cells producing autoantibodies against neuronal and myelin antigens (Hayes et al., 2002; Ankeny et al., 2006, 2009).

### 2.3. Compartmentalization of the SCI lesion site

**FIGURE 2:**
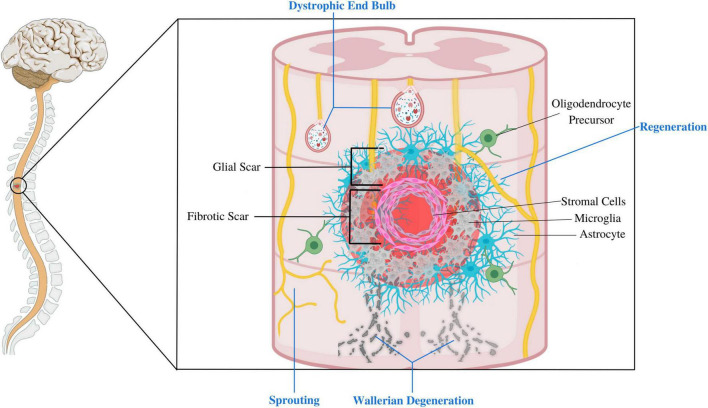
This figure depicts the composition of different compartments of the SCI lesion site. The fibrotic scar mainly comprises stromal cells, blood-borne inflammatory cells, and microglia. This core is surrounded by glial scar, composed of an astrocyte limitans border, oligodendrocyte progenitor cells (OPCs), and microglia. Extensive reactive changes also take place in the adjacent neural parenchyma, including axonal dieback and synaptic remodeling and axonal sprouting of spared axons. Glial cells such as astrocytes, OPCs and microglia in this peri-lesional area are reactive, but differ in their magnitude of reactivity than those at the lesion site. This figure was created with Biorender.com.

Recent research advocates dividing the SCI scar into three compartments: the inner fibrotic scar, the surrounding astroglial border (or glial scar), and the adjacent reactive neural parenchyma (Figure 2; O’Shea et al., 2017). These compartments exhibit unique cellular compositions and transcriptional profiles (Gong et al., 2023). The central fibrotic scar consists of macrophages, other blood-borne inflammatory cells like lymphocytes, and stromal cells such as fibroblasts and pericytes. Over time, blood-borne inflammatory cells recede (albeit not completely), leaving stromal elements to constitute the bulk of the fibrotic scar (Beck et al., 2010). The astrocyte border comprises proliferating astrocytes up until 14 dpi, after which the structure matures. NG2^+^ OPCs are also found in this region (Keirstead et al., 1998; Miron et al., 2013). The adjacent reactive neural parenchyma comprises neurons that display active synaptic remodeling and circuit reorganization. Glial cells in this region are composed of reactive astrocytes, microglia, and OPCs, but they differ in their magnitude of reactivity from their counterparts in the lesion core and border (Khakh and Sofroniew, 2015). For example, astrocytes outside the lesion site upregulate glial-fibrillary acid protein (GFAP) but do not dramatically change their morphology and orientation and can even stimulate the regeneration of adult CNS neurons (Davies et al., 1999; Li X. et al., 2020). In contrast, astrocytes within the scar border more drastically upregulate GFAP and significantly change their morphology and orientation to form a compact glial scar that impedes regeneration (Tran et al., 2018b).

It is essential to state that the term “glial scar” was traditionally used to refer to the whole SCI scar. This is now considered a misnomer by prominent researchers as it carries negative connotations that depict the glial scar only as an obstacle to axon regeneration and functional recovery. This oversimplification disregards the dynamic and multifaceted nature of the host response to SCI (Adams and Gallo, 2018; Sofroniew, 2020; Wahane and Sofroniew, 2022). Adopting accurate terminology by describing the different compartments and time-dependent roles of the SCI scar allows for a more nuanced understanding of the SCI response, particularly for non-expert readers. Using broad or general terms like “glial scar” to refer to the SCI lesion site perpetuates the misconception that the SCI scar is inherently inhibitory and requires wholesale/indiscriminate attenuation (Wahane and Sofroniew, 2022), which may hinder the development of effective therapeutic strategies targeting the diverse functions of glial and stromal cells. Unfortunately, there is still no consensus regarding the correct use of the term “glial scar” or agreed-upon alternate terms. In this review, we adopt the terminology used by Adams and Gallo (2018), who used “glial scar” to refer to the glial cell border—composed of astrocytes and OPCs—surrounding the fibrotic core of the lesion.

### 2.4. Extracellular regeneration inhibitors in the glial scar

Myelin’s role as a CNS axonal regeneration inhibitor was first described in the 1980s (Schwab and Strittmatter, 2014). Myelin-associated molecules inhibiting CNS regeneration include Nogo (reticulon-4), Nogo-receptors (NgR), myelin-associated glycoprotein (MAG), and oligodendrocyte myelin glycoprotein (OMgp) (Bradbury and McMahon, 2006). While experiments in the early 2000s found that systemic or local administration of anti-Nogo receptor antibodies enhanced CNS regeneration (Silver and Miller, 2004; Lu et al., 2012), others utilizing genetic knockout (KO) of myelin-associated molecules did not consistently reproduce these results (Anderson et al., 2018; Liu et al., 2022). This discrepancy suggests that myelin may not play a significant role in CNS regeneration failure, but that inhibiting myelin-associated molecules could enhance the remodeling of spared axons to provide functional improvement (Liu et al., 2022). The RESET trial, completed on June 2022, is a two-part clinical trial studying AXER-204, a human fusion protein that functions as a decoy for myelin-associated inhibitors Nogo-A, MAG, and OMgp (NCT03989440).

Chondroitin sulfate proteoglycans (CSPGs) are produced by various cell types, including astrocytes, microglia, fibroblasts, pericytes, and OPCs, and play a role in inhibiting axon growth (Gallo et al., 1987; Tran et al., 2018b). CSPGs bind to the surface receptor protein tyrosine phosphatase-σ (RPTPσ) on neurons, disrupting neuronal autophagy and leading to axon growth cone dystrophy and regeneration failure (Shen et al., 2009). Strategies to counteract CSPGs include administering chondroitinase ABC to digest glycosaminoglycan (GAG) side chains, preventing CSPG formation, or abrogating RPTPσ signaling (see Tran et al., 2018a,b for a detailed review). These approaches reproducibly alleviate axon growth inhibition and promote some functional recovery (Shen et al., 2009; Lang et al., 2015; Rink et al., 2018) but do not yield significant regeneration benefits when used alone (Zheng and Tuszynski, 2023). Furthermore, since aggrecan—the prototypical CSPG—is mainly present in perineuronal nets (PNN) and to a lesser extent in the core of SCI lesions, it appears that inhibiting CSPG function enhances axon sprouting and neuronal plasticity in the reactive CNS parenchyma rather than directly allowing axon regrowth through the SCI scar (Fawcett, 2015). In this regard, chondroitinase ABC reduces the atrophy of spared corticospinal tracts after SCI and promotes axonal sprouting and circuit reorganization (Carter et al., 2008; Starkey et al., 2012). From these results, the future role of extracellular inhibition strategies will likely be limited to combinatorial approaches supplementing neuron-intrinsic regeneration strategies such as NSC transplantation or exogenous delivery of growth factors. Detailed reviews on the role of perineural nets and CSPGs in SCI are referenced here (Tran et al., 2018b,^2022^).

### 2.5. The SCI scar and spinal cord regeneration: revisiting historical misconceptions

Throughout the 20th century, studies found that injured CNS axons regrew through living peripheral nerve grafts but not CNS white matter, indicating that the CNS microenvironment might lack certain growth-promoting and/or contain growth-inhibitory factors (Sugar and Gerard, 1940; Brown and McCouch, 1947; Richardson et al., 1980; Silver and Miller, 2004; Anderson et al., 2016). Additionally, the observation of dystrophic end-bulbs of non-regenerating axons abutting the astroglial border led to the hypothesis that the SCI scar formed a physical barrier impeding axonal regeneration (Silver and Miller, 2004).

However, rigorous testing in the past two decades has shown that the SCI scar plays both beneficial and harmful roles in SCI. For instance, loss-of-function experiments in transgenic mice have revealed a protective role of acute glial and stromal cell responses in SCI (Table 1). It is now clear that the SCI scar is a double-edged sword: it is acutely beneficial by performing damage-containment functions that prevent the propagation of the primary injury but, in the long-term, contributes to spinal cord regeneration failure by virtue of its extracellular inhibitors, physically insurmountable nature, and continual pro-inflammatory cellular signatures (Silver and Miller, 2004; Gaudet and Fonken, 2018; Escartin et al., 2021).

**TABLE 1:** Genetic or pharmacologic loss-of-function experiments have revealed vital neuroprotective functions of glial and stromal responses to SCI.

Cell type	Reference	Loss of function model	Impact on SCI outcome
Astrocytes	Faulkner et al., 2004	TK+ GCV	Failure of wound contraction Prolonged neuronal loss Persistent neurological deficits
Astrocytes	Anderson et al., 2016	TK+ GCV, *stat3*-cKO	Failure of axonal regrowth Significant increase in axonal dieback
Astrocytes	Wanner et al., 2013	*Stat3*-cKO	Poorly demarcated scar border with extensive spread of inflammatory cells
Microglia	Bellver-Landete et al., 2019	PLX5622	Impaired locomotor recovery following SCI Impaired astrocyte proliferation Decreased survival of neurons and oligodendrocytes at site of injury
Microglia	Zhou X. et al., 2020	*Plxnb2*-cKO	Significant impairment in sensorimotor recovery Impaired wound compaction leading to enlargement of SCI lesion
OPC	Hesp et al., 2018	NG2 TK+ GCV	Enlarged lesions with edema Prolonged hemorrhage Inhibition of angiogenesis at site of lesion
OPC	Bartus et al., 2019	*ErbB*-cKO	Impaired locomotor recovery
Stromal cells	Yokota et al., 2017	*Postn*-KO	Decreased fibrotic scar formation Impaired functional recovery

### 2.6. Spinal cord regeneration across different phyla

Spinal cord injury biology research has revealed the remarkable regenerative capacity of invertebrates and several non-mammalian vertebrates, such as Zebrafish, Urodeles (newts and salamanders), Lamprey, and Xenopus frogs (Tran et al., 2022). These animals form a glial bridge across the SCI lesion site, regenerate their spinal cords without scar formation, and spontaneously return to full autonomic and sensorimotor function (Ferretti et al., 2003; Shifman et al., 2006; Tazaki et al., 2017; Ghosh and Hui, 2018; Freitas et al., 2019; Sabin et al., 2019; Tsata and Wehner, 2021). These studies also emphasize the hostile and growth-inhibitory nature of the SCI scar in mammals, as regeneration in amphibians begins to fail when scar tissue forms (Bertolotti et al., 2013; Edwards-Faret et al., 2021). Scarring after injury appears to be a phenomenon acquired during evolution that impairs spinal cord regeneration after injury.

Previously, excellent spinal cord regenerative capabilities were considered unique to invertebrates and lower, non-mammalian vertebrates. However, new data shows that adult spiny mice (*Acomys cahirinus*) can spontaneously recover complete bladder control after spinal cord transection at the T8 vertebral level (Nogueira-Rodrigues et al., 2022). Although locomotor recovery in adult spiny mice remains incomplete, it still far exceeds any magnitude of recovery observed in adult mammals after complete SCI (Nogueira-Rodrigues et al., 2022). Therefore, a mammalian adult spinal cord regeneration model is now available (Gaire et al., 2021; Wehner and Becker, 2022).

### 2.7. Spinal cord regeneration throughout lifespan

Age is a crucial factor in determining spinal cord regenerative capacity in mammals. Neonatal mice (post-natal day 2) and the prematurely born South American opossum (*Monodelphis domestica*) can robustly regenerate their spinal cords without scar formation, reintegrate with distal neural circuitry, and recover neurologic function (Mladinic and Wintzer, 2002; Li X. et al., 2020). However, scarring occurs globally in 7-day-old mice and 2–3-week-old opossums, with a consequent loss in their regeneration capacity (Mladinic and Wintzer, 2002; Li X. et al., 2020).

The decline in mammalian CNS regenerative capacity in the post-natal period is multi-factorial. One reason is the significant change in the proteome of CNS neurons with age (Agrawal and Welshhans, 2021), which likely impacts post-injury regenerative capacity (Tran et al., 2022). During the embryonic period, CNS neurons are programmed to grow far-reaching axons to reach distal innervation targets, while in post-embryonic life, they facilitate and maintain local synaptic plasticity (Gumy et al., 2011; Shigeoka et al., 2016; Agrawal and Welshhans, 2021). For example, the alpha2delta2 subunit of voltage-gated calcium channels (VGCCs) is expressed during late embryogenesis, acting as a developmental switch that inhibits the axonal growth characteristic of early developmental CNS axons (Tedeschi et al., 2016). Genetically deleting the Cacna2d2 gene, which encodes the alpha2delta2 subunit, promotes axonal growth *in vitro* and inhibiting the alpha2delta2 subunit with pregabalin in adult mice after SCI enhances axonal regeneration (Tedeschi et al., 2016). The specific regulators of this developmental switch are yet to be fully understood. Still, they likely involve combined effects from various extracellular factors, including CSPG-RPTPσ signaling and possibly astrocyte-derived synaptogenic signals like thrombospondins, which upregulate alpha2delta1 subunit of VGCCs on neurons (Christopherson et al., 2005; Risher and Eroglu, 2012; Brooks et al., 2013; Sakamoto et al., 2019; Tran et al., 2020).

Embryonic neurons are less sensitive to inhibition by CSPGs and myelin-derived components like Nogo-A than adult neurons (Carulli et al., 2005; See et al., 2010; Poplawski et al., 2020). The concentration of these extracellular inhibitors is also much lower within the neonatal SCI lesion site than in adults (Tran et al., 2022). Recent novel findings by Nogueira-Rodrigues et al. showed that the extraordinary regenerative capacity of adult *Acomys* after SCI was underpinned by a pro-regenerative ECM signature. In their study, the SCI microenvironment of *Acomys* was highly enriched in keratin sulfate proteoglycans (KSPGs) and β3gnt7, the enzyme involved in KSPG production (Nogueira-Rodrigues et al., 2022). β3gnt7-expressing cells promoted neurite outgrowth in vitro, attributing growth-stimulating properties to KSPGs (Nogueira-Rodrigues et al., 2022). It would be interesting to investigate if experimentally engineering the ECM signature toward KSPG predominance in animal models known for poor spinal cord regeneration can augment axonal regeneration. Prior research has explicitly shown that KSPGs limit neuronal plasticity in rats, and their degradation by keratanase-II improves sensorimotor recovery after SCI, demonstrating comparable efficacy to chondroitinase ABC (Imagama et al., 2011). The factors driving these divergent responses to KSPGs in *Acomys* spiny mice and rats are yet to be elucidated. It would also be worthwhile investigating whether regenerative animal models that display scarless healing exhibit a core, pro-regenerative ECM composition.

The non-neuronal cellular response to injury also varies between embryonic and post-natal life. For example, immature astrocytes react less severely to stimuli like amyloid-β than mature astrocytes (Rudge and Silver, 1990; Canning et al., 1993). When reactive, immature astrocytes demonstrate reduced hypertrophy than their adult counterparts and are less densely packed at the scar border. Such an arrangement allows them to retain essential wound-sealing functions and creates an environment more favorable for axonal regeneration (Smith et al., 1987; Balasingam et al., 1994; Domowicz et al., 2011). After perinatal ischemic stroke, immature reactive astrocytes elaborate neuroprotective factors, including PGDF, IGF, and VEGF (Revuelta et al., 2019). These beneficial effects are most apparent when implanting immature astrocytes into adult SCI lesions, which leads to reduced glial scarring, enhanced axonal growth, and improved functional recovery (Davies et al., 2006, 2011; Filous et al., 2010; Haas and Fischer, 2013). In contrast, transplanting mature astrocytes impairs healing by recruiting macrophages and fibroblasts, resulting in cavitation (Filous et al., 2010).

Groundbreaking findings from Li Y. et al. (2020) demonstrated that microglia in neonatal mice populate the injury site, generating fibronectin and protease inhibitors that connect severed axon ends and enable scar-free axonal repair. Adult microglia only transiently and partially recapitulate the gene expression profile of their neonatal counterparts (Li Y. et al., 2020; Wahane et al., 2021), which can drive proliferation, revascularization, and functional recovery (Wang et al., 2022). However, adult microglial subsets displaying these developmental, pro-regenerative signatures are significantly less abundant in adult SCI lesion sites and overexpress and under-express CD68 and P2ry12, respectively, which may diminish their regenerative capacity (Li et al., 2022). Determining the factors that drive this developmental gene signature in immature astrocytes and microglia and how it can be augmented in their adult counterparts is a critical area for future research.

Above, we have provided compelling evidence that spinal cord regenerative capacity varies considerably between embryonic and post-natal life. As aging research has garnered a massive rise in interest in recent years, studies have also shown that older patients display poorer neurologic outcomes after SCI compared to younger individuals (Scivoletto et al., 2003; Furlan and Fehlings, 2009), suggesting the existence of a “second wave” of changes in the CNS injury response that subjects older individuals to a greater neuropathologic burden and worse clinical outcomes. López-Otín et al. (2013; 2023) identified twelve biological aging hallmarks: genomic instability, telomere shortening (i.e., attrition), epigenetic alterations, loss of proteostasis, dysregulated nutrient sensing, mitochondrial dysfunction, stem cell exhaustion, altered intercellular communication, macroautophagy, chronic low-grade inflammation, and gut microbiome dysbiosis. The geroscience hypothesis states that the accumulation of these processes in different body tissues—at different rates—drives aging-related tissue dysfunction and that targeting biological aging processes may extend healthspan and potentially even lifespan (Kennedy et al., 2014). The accumulation of biological aging hallmarks is evident in the CNS and manifests as age-related changes in cerebral morphology, impaired neurogenesis, and neuroinflammation (see Gonzales et al., 2022 for a detailed review).

Cellular senescence, a state of irreversible cell cycle arrest accompanied by characteristic molecular, morphological, and functional alterations, has emerged as a key therapeutic target in aging and chronic diseases, including neurodegenerative diseases (Kirkland et al., 2017; Kirkland and Tchkonia, 2017, 2020; Hernandez-Segura et al., 2018; Calcinotto et al., 2019; Gorgoulis et al., 2019; Gasek et al., 2021; Chaib et al., 2022; Huang W. et al., 2022; Shafqat et al., 2022; Zhang L. et al., 2022). Senolytics, which are drugs that eliminate senescent cells, are already being evaluated in clinical trials for a host of chronic diseases such as Alzheimer’s disease (see Zhang L. et al., 2022 for a detailed review).

Hence, recent studies have defined the role of cellular senescence in the aging CNS in an attempt to uncover novel therapeutic targets. Pericytes in the aging brain undergo senescence, associated with increased blood-brain barrier (BBB) permeability *in vitro*, suggesting that senescent pericytes could partially contribute to age-related BBB dysfunction and neuroinflammation (Iwao et al., 2023). Senescent microglia accumulate in the aged brain and elaborate pro-inflammatory cytokines and chemokines that recruit adaptive T-cells and B-cells, which are linked to the onset of cognitive decline (Ogrodnik et al., 2021; Zhang X. et al., 2022). The pro-inflammatory phenotypes of senescent microglia can be augmented by stressors such as traumatic brain injury (TBI), resulting in more pronounced neuroinflammatory responses in older mice and worse cognitive outcomes than in younger mice (Ritzel et al., 2019). Lastly, adult neurogenesis occurs in the dentate gyrus of the hippocampus but decreases with age due to a decrease in the number of NSCs and neuroblasts, connected to the onset of age-related cognitive decline (Kase et al., 2020). A recent study demonstrated an accumulation of senescent neuroblasts in the dentate gyrus with aging (Jin et al., 2021). These neuroblasts release pro-inflammatory molecules that recruit natural killer (NK) cells, subsequently eliminating senescent neuroblasts, leading to impaired neurogenesis and cognition (Jin et al., 2021).

It is curious, perhaps even paradoxical that cellular senescence is an evolutionarily conserved phenomenon despite its adverse effects on nearly every organ system. However, senescence has crucial beneficial roles in embryogenesis and is a frontline defense against tumorigenesis (Storer et al., 2013; Lorda-Diez et al., 2015; Schosserer et al., 2017). Cellular senescence exerts both beneficial and harmful effects on wound healing. Pioneering work from Demaria et al. (2014) demonstrated that—in p16-3MR mouse models that allow tracing and inducible depletion of senescent cells—fibroblasts and endothelial undergo senescence early after a cutaneous wound. These senescent cells secrete PDGF-AA, stimulating the differentiation of local fibroblasts to myofibroblasts that mediate wound contraction (Demaria et al., 2014). Depleting senescent cells in transgenic mice delayed wound healing (Demaria et al., 2014). However, successful wound healing requires the clearance of senescent fibroblasts and endothelial cells, as their persistent accumulation drives inflammation and tissue dysfunction via their senescence-associated secretory phenotype (SASP) (Childs et al., 2015; Calcinotto et al., 2019). Similarly, experiments analyzing the injury response in the zebrafish hearts and fins and salamander limbs reveal a transient induction of cellular senescence that, if disrupted, impairs the regenerative response (Yun et al., 2015; Da Silva-Álvarez et al., 2020).

Paramos-de-Carvalho et al. (2021) conducted a comparative study on the dynamics of cellular senescence in SCI between zebrafish and adult mice. They discovered that SCI upregulates senescence-associated β-galactosidase (SA β-gal), the most widely used marker for senescent cells, in neurons at the lesion periphery in zebrafish and mice. Striking differences were observed in the temporal dynamics of senescent neurons: in zebrafish, the number of senescent neurons peaked at 8.9% at 15 dpi but then steadily declined to reach baseline levels by 60 dpi, in line with the idea that transient senescence induction is a conserved process associated with successful wound healing and regeneration (Paramos-de-Carvalho et al., 2021). Conversely, in mice, the percentage of total senescent neurons was 25.3% at 15 dpi and continued to increase until 60 dpi, reaching 35.3% (Paramos-de-Carvalho et al., 2021). When the mice were treated with ABT-263, a known senolytic, they exhibited significantly better sensorimotor and bladder function recovery than vehicle-treated mice, indicating that the accumulation of senescent cells contributes to the growth-inhibitory SCI microenvironment in mice. The functional recovery was associated with increased white matter sparing and enhanced synaptic plasticity in the adjacent reactive neural parenchyma (Paramos-de-Carvalho et al., 2021). To test the hypothesis that chronic senescent accumulation in non-healing wounds promotes inflammation, the authors demonstrated that ABT-263 significantly reduced inflammatory macrophages numbers and levels of pro-inflammatory cytokines, chemokines, and mitogenic and fibrogenic growth factors in the SCI scar (Paramos-de-Carvalho et al., 2021).

## 3. Astrocytes

Astrocytes are of neuroectodermal origin and constitute about 20% of glial cells (Molofsky and Deneen, 2015; Khakh and Deneen, 2019). They fulfill diverse physiological roles in the CNS, including blood-brain barrier (BBB) maintenance, neurotransmitter uptake for synapse homeostasis, energy substrate provision to neurons, and interactions with other astrocytes, oligodendrocytes, and microglia (Khakh and Deneen, 2019). Following CNS injury, astrocytes become reactive, which entails an array of molecular, morphological, and functional alterations that impact adjacent cells, positively or negatively, depending on the disease context (Sofroniew and Vinters, 2010; Sofroniew, 2020). This process is often incorrectly termed astrogliosis, which entails astrocyte proliferation. Reactive astrogliosis constitutes a small portion of the reactive astrocyte response at the SCI lesion penumbra. Rather, much of the reactive astrocytic response consists of morphologic alterations collectively referred to as reactive astrocytosis, including hypertrophy of astrocytic processes, a consequent overlap between spatially defined astrocyte domains, and cytoskeletal rearrangements such as upregulation of the intermediate filaments GFAP and vimentin (Daniel et al., 2010).

### 3.1. Historical perspective

Reactive astrocytes were once considered the primary contributors to post-SCI regeneration failure by creating a physical barrier and producing inhibitory CSPGs (Silver and Miller, 2004). This belief was supported by histological evidence depicting dystrophic axon end-bulbs abutting the astrocyte limitans border (Aguayo et al., 1981; David and Aguayo, 1981; Schwab and Bartholdi, 1996; Davies et al., 1997). Thus, researchers hypothesized that depleting astrocytes or key signaling pathways using transgenic models would enhance axonal regeneration across the scar (Table 1).

### 3.2. Beneficial astrocyte reactivity

Astrocyte biology in SCI proved more complex than initially assumed. Transgenic ablation of astrocytes, disruption of astrocyte scar-forming function, or reducing the number of border-forming astrocytes does not improve the regeneration of transected corticospinal, sensory, or serotonergic axons (Anderson et al., 2016). Such manipulations exacerbate neuroinflammation and neuronal loss (Bush et al., 1999; Faulkner et al., 2004; Myer et al., 2006; Gu et al., 2019; Zhao W. et al., 2022). Therefore, scar-forming astrocytes do not acutely inhibit axonal growth; instead, they recruit inflammatory cells to the lesion epicenter and then proliferate to seal it off, confining neuroinflammation (Wanner et al., 2013; Sofroniew, 2015). Specific subsets of activated astrocytes may even mitigate neuroinflammation outgrowth in SCI by upregulating anti-inflammatory molecules like clusterin (Wright et al., 2014; De Miguel et al., 2021; Gong et al., 2023).

Border-forming astrocytes can promote the growth of maximally stimulated axons by producing integrin, which binds axon growth cones and enhances their growth (Anderson et al., 2016). Similarly, yes-associated protein (YAP), which contributes to the exceptional regenerative abilities of lower non-mammalian vertebrates like zebrafish, is upregulated in mouse astrocytes by basic fibroblast growth factor (bFGF) in the SCI microenvironment and promotes astrocyte proliferation, protective glial scar formation, axonal regeneration, and functional recovery (Xie et al., 2020; Riley et al., 2022). A recent study transplanted anti-inflammatory/pro-repair astrocytes to promote axonal regeneration, remyelination, and functional recovery after SCI (Chang et al., 2023). Astrocytes have recently been converted into neurons to aid synaptic remodeling and functional recovery (Su et al., 2014; Noristani et al., 2016; Puls et al., 2020).

An essential question is how to reconcile these apparent axon growth-enhancing effects of scar-forming astrocytes with their propensity to produce inhibitory CSPGs. Hypertrophic astrocytes in the surrounding CNS parenchyma elaborate CSPGs to influence local synaptic remodeling (O’Shea et al., 2017; Sofroniew, 2018; Santello et al., 2019). This explains why modulating astrocyte-derived CSPGs can augment functionally beneficial synaptic remodeling proximal to the lesion site (discussed above in the section “*Extracellular Regeneration Inhibitors in the Glial Scar”*). Furthermore, recent studies have demonstrated that astrocytes are not the primary source of inhibitory CSPGs, mainly derived from stromal cells, OPCs, and macrophages (Jones et al., 2002; Anderson et al., 2016).

### 3.3. Dysfunctional astrocyte reactivity

While acknowledging the evidence discussed earlier, it is crucial to recognize that particular astrocyte responses can be detrimental, referred to as dysfunctional astrocyte reactivity.

Dysfunctionally reactive astrocytes can promote BBB disruption and neuroinflammation through TNF-STAT3 signaling and alpha-1-antichymotrypsin production (Kim et al., 2022). Additionally, pro-inflammatory cytokines derived from microglia foster pro-inflammatory and neurotoxic reactive astrocyte phenotypes linked to the pathogenesis of neurodegenerative diseases (Phatnani and Maniatis, 2015; Liddelow et al., 2017; Russ et al., 2021; Brandebura et al., 2023). Amyloid-β was also recently shown to directly provoke pro-inflammatory and neurotoxic astrocyte reactivity, leading to synaptic and neuronal loss (Jiwaji et al., 2022). Reactive astrocytes can increase the expression of genes encoding proteins like thrombospondins, which facilitate synaptogenesis (Christopherson et al., 2005; Risher and Eroglu, 2012; Risher et al., 2018). However, thrombospondins may also lead to the formation of unwanted synapses, leading to epilepsy or neuropathic pain (Boroujerdi et al., 2008; Liddelow and Barres, 2017; Cui et al., 2021).

In SCI, it is still true that the chronic presence of the densely packed astroglial scar constitutes a physical barrier to axonal regeneration. The formation of the astroglial scar depends on microenvironmental signals within the injured spinal cord, as Hara et al. (2017) elegantly demonstrated. Astrocytes elicit reactive gliotic responses when transplanted into the injured spinal cord but revert to quiescent, non-reactive states when transplanted into a naïve spinal cord (Hara et al., 2017). In the injured spinal cord, type I collagen partly facilitates the dense packing of astrocytes through the integrin/N-cadherin signaling pathway (Hara et al., 2017). Attenuating integrin signaling reduces astroglial scarring—but does not deplete astrocytes—and leads to improved axonal regrowth and functional recovery (Kanemaru et al., 2013; Hara et al., 2017). Other studies have similarly demonstrated that carefully manipulating astrocyte functions rather than all-or-none genetic or pharmacologic ablation techniques can “loosen” the astrocyte scar and augment neuronal and functional recovery (Iseda et al., 2004; Ma et al., 2004; Hurtado et al., 2011). The severity of SCI adds another layer of complexity to the dual role of astrocytes: milder forms of injury lack the dense macrophage and stromal cell infiltrate and feature lower levels of ECM elaboration, which can reprogram astrocytes to promote neurite outgrowth and axonal regeneration (Fitch and Silver, 2008; Alicia et al., 2011; Silver, 2016). More severe injuries elicit robust GFAP upregulation and dense, growth-blocking scar formation (Fitch and Silver, 2008; Alicia et al., 2011; Silver, 2016).

Astrocytic SOSC3 signaling plays a role in glial scarring and diminished functional recovery after SCI, while attenuating SOCS3 reduces scarring and promotes remyelination and functional recovery (Okada et al., 2006; Hackett et al., 2016). Similarly, the upregulation of erythropoietin-producing hepatocyte A4 (EphA4) on neurons post-SCI binds to ephrin-B receptors on astrocytes, inducing pro-inflammatory astrocyte reactivity, which hinders neurite outgrowth and axonal regeneration (Chen et al., 2022). Genetic ablation of the ephrin-B receptor on astrocytes leads to improved axonal regeneration following SCI (Chen et al., 2022). Epigenetic regulation by several micro-RNAs has also been widely implicated in stimulating the hypertrophy and proliferation of reactive astrocytes, promoting glial scar formation (Liu R. et al., 2018). For example, a recent study showed that microRNA mir-155-5p stimulates astrocyte proliferation and inhibits their apoptosis after SCI, facilitating reactive astrogliosis and scar formation (He et al., 2023). Silencing mir-155-5p decreases GFAP and NF-200 expression and attenuates astroglial scar formation, which is associated with better locomotor recovery in mice (He et al., 2023).

To conclude, reactive astrocytosis and astrogliosis are beneficial in the acute and subacute phases of SCI, serving to contain neuroinflammation. However, the formation of a dense astroglial scar in the chronic phase of SCI constitutes a physical barrier to axonal regeneration. Moreover, the upregulation of particular signaling pathways in scar-forming astrocytes can obstruct axonal regeneration and functional recovery after SCI, and targeting these regulators may have future clinical applications in promoting axonal regeneration.

### 3.4. Astrocyte heterogeneity

Astrocytes are a diverse group of cells that exert region-dependent functions in the healthy CNS and differentially modulate local neuronal circuitry (Tsai et al., 2012; Matias et al., 2019; Huang et al., 2020). The heterogeneity of astrocytes has become a key focus in neuroscience research.

Astrocytes mount context-specific responses to CNS injuries (Yu et al., 2020). For example, profiling astrocyte transcriptomes by microarray or single-cell RNA sequencing (scRNA-Seq) in stab wound injury, lipopolysaccharide (LPS)-induced neuroinflammation, ischemic stroke, SCI, and neurodegeneration reveals disease-specific gene expression (Zamanian et al., 2012; Liddelow et al., 2017; Cao et al., 2022). Liddlelow et al. categorized transcriptionally distinct astrocyte subsets into “A1” and “A2,” with opposing effects in various disease states: A1 astrocytes are pro-inflammatory and neurotoxic, whereas A2 astrocytes promote tissue repair and are neuroprotective. However, the functions of A1 and A2 genes are largely unknown, and astrocytes often display a mix of A1/A2 gene signatures in CNS disease (Grubman et al., 2019; Al-Dalahmah et al., 2020; Das et al., 2020; Zhou Y. et al., 2020), leading researchers to recommend moving past the binary A1/A2 classification (Escartin et al., 2021). Nonetheless, it remains that transcriptional astrocyte diversity can foster either dysfunctional astrocyte reactivity that promotes neuropathology or resilient reactive states that support wound resolution and functional recovery (Liddelow et al., 2017; Wheeler et al., 2020; Yang et al., 2020a). Multiple sclerosis research has shown that both dysfunctional and resilient populations of astrocytes can coexist and vary with disease stage (Wheeler and Quintana, 2019; Wheeler et al., 2020). Similarly, amyloid-β and hyperphosphorylated tau induce pathologic and protective astrocyte phenotypes, respectively, suggesting that both populations co-exist in Alzheimer’s disease (Jiwaji et al., 2022). Notably, the same transcriptional regulators (TRs) can have protective or detrimental roles depending on the disease context. For example, STAT3 signaling is neuroprotective in TBI (Nobuta et al., 2012) and SCI (Herrmann et al., 2008; Wanner et al., 2013) but harmful in Alzheimer’s disease (Ceyzériat et al., 2018; Reichenbach et al., 2019).

Astrocyte heterogeneity in SCI has been investigated as well. White and colleagues used immunohistochemical staining to reveal morphological differences among astrocytes in the cervical, thoracic, and lumbar spinal segments of a contusive SCI mouse model (White et al., 2010). ScRNA-Seq showed that reactive astrocytes in SCI exhibit a substantially different transcriptome, sharing only partial similarities with the steady-state CNS and other CNS disorders (Burda et al., 2022).

Hou et al. (2022) recently employed scRNA-Seq to identify 12 transcriptionally distinct clusters of astrocytes following traumatic SCI. By using Gene Ontology (GO) enrichment analysis, KEGG pathway analysis, and the use of “A1/A2” marker genes, the authors inferred that each of the 12 clusters had uniquely enriched genes, possibly pointing to distinct roles in SCI (Hou et al., 2022). Moreover, each cluster exhibited differential temporal dynamics within the SCI lesion site: “A1” astrocyte clusters were most abundant in the acute and subacute phases, whereas “A2” reactive clusters (Silver and Miller, 2004; Adams and Gallo, 2018; Orr and Gensel, 2018; Liu et al., 2022) were more abundant in the subacute and chronic phases (Hou et al., 2022). This study also identified several biomarkers that may facilitate cluster-specific manipulation experiments to test whether enhancing “A2” subsets or inhibiting “A1” reactive astrocytes may improve post-SCI neural regeneration and functional recovery (Hou et al., 2022).

However, inferring functional states from gene expression data can be misleading since the transcriptional analysis does not always accurately reflect functional activity, especially in the highly dynamic *in vivo* environment and given the complexity of cellular interactions. Ultimately, only loss-of-function experiments targeting essential proteins enriched in different astrocyte subsets will causally link molecular heterogeneity to function. Also, as discussed above, the “A1/A2” terminology is now considered outdated.

To address how context-specific astrocyte reactivity is regulated, the Sofroniew Laboratory used scRNA-Seq and transcriptional regulator enrichment analysis (TREA) followed by numerous validation techniques to predict TRs of disease-specific astrocytic reactivity in SCI, LPS-induced neuroinflammation, and experimental autoimmune encephalomyelitis (EAE), which is a mouse model of multiple sclerosis (Burda et al., 2022). Strikingly, genetic *KO* models of key TRs such as Smarca4 and Stat3 showed that they could regulate the same differentially expressed gene oppositely (e.g., Stat3 and Smarca4 can upregulate *Slc14a2* and *Rhof* in LPS and downregulate them in SCI), which better contextualizes findings highlighting divergent functions of Stat3 in different CNS diseases (discussed above) (Burda et al., 2022). The identified TRs, including Stat3 and Smarca4, were shown to influence disorder outcome, as their genetic deletion worsened SCI neuropathology and functional outcomes in mice, suggesting that targeting these TRs could have future clinical applications (Burda et al., 2022). Lastly, although over 10,500 differentially expressed genes were identified in astrocytes across eight CNS disorders with little overlap between diseases, a core of 61 astrocyte reactivity TRs were shared in at least 7 of 8 conditions, including SCI (Burda et al., 2022).

These data suggest that a limit number of TRs exert a combinatorial control over reactive astrocyte gene expression to achieve remarkably heterogeneous context-specific astrocyte responses that influence disease outcomes. Elucidating the extrinsic modulators of core TRs and the astrocyte functions promoted by each TR could reveal translational opportunities to mitigate dysfunctionally reactive astrocytes and/or enhance resilient reactive subsets. For instance, a recent study demonstrated that exogenously delivering the TR Sox2 reprogrammed astrocytes into a pro-regenerative phenotype, which promoted axonal regeneration, reduced dense glial scarring, and enhanced functional recovery when combined with rehabilitation strategies that improve neuronal plasticity (Yang et al., 2020b).

## 4. Microglia

Microglia are the resident immune cells of the CNS, originate from the yolk sac, and perform various functions in both healthy and diseased CNS states. In the healthy CNS, microglia are non-motile but extend highly dynamic processes that survey the extracellular environment to carry out “housekeeping” functions, including phagocytosing extracellular debris or pathogenes, fortifying the BBB, delivering nutritional support to neurons and oligodendroglia, orchestrating synaptic pruning, and sustaining myelin turnover (Paolicelli et al., 2011; Schafer et al., 2012; Domingues et al., 2016; Haruwaka et al., 2019; Hughes and Appel, 2020; Ronaldson and Davis, 2020; Santos and Fields, 2021; McNamara et al., 2023).

### 4.1. Microglia heterogeneity

Microglia exhibit distinct morphological and functional properties in the healthy CNS, contingent upon their location, ontogeny/developmental origin, and local microenvironmental signals such as astrocyte-derived cytokines/chemokines (Bennett et al., 2018; Zheng et al., 2021; Lynch, 2022). This heterogeneity wanes with aging, but specific location-dependent differences in microglial identity are maintained in the adult CNS (Grabert et al., 2016; Masuda et al., 2019). Furthermore, sex-specific differences in microglial morphology, transcriptome, and proteome have been identified, potentially contributing to gender-related variation in CNS disease pathophysiology and manifestations (Guneykaya et al., 2018; Lynch, 2022).

Heterogeneity intensifies when considering CNS-resident macrophages or border-associated macrophages (BAMs), which reside within the meninges, choroid plexus, and perivasculature. These BAMs are transcriptionally and functionally unique from microglia (Zeisel et al., 2015; Mrdjen et al., 2018; Van Hove et al., 2019; Prinz et al., 2021; Masuda et al., 2022). However, their distinct roles in SCI remain largely uncharted (Jordão et al., 2019; Kierdorf et al., 2019; De Schepper et al., 2023). Additionally, under specific circumstances such as ischemic stroke, pericytes can differentiate into microglia- and macrophage-like cells (Nirwane and Yao, 2022), introducing another facet of microglial heterogeneity.

Functionally, microglial and macrophages have traditionally been categorized into “M1” (pro-inflammatory, cytotoxic) or “M2” (anti-inflammatory, pro-repair) (David and Kroner, 2011). This is an *in vitro* classification derived from experiments that show that stimulating macrophages with IFN-γ, TNF-α, or LPS induces macrophages toward pro-inflammatory cytokine production, whereas IL-4 or IL-13 polarize macrophages toward the production of anti-inflammatory cytokines (Gordon, 2003; David and Kroner, 2011). However, the *in vivo* microenvironment contains a mix of “M1-favoring” and “M2-favoring” DAMPs, cytokines, and chemokines (Xue et al., 2014). Moreover, the SCI environment is highly dynamic in that the balance between these factors constantly changes. Hence, neatly categorizing microglial and macrophage activation states into M1 and M2 does not reflect the *in vivo* reality (David et al., 2018). Contemporary research has unveiled that microglia and macrophages exhibit mixed M1/M2 gene signatures upon activation (Kigerl et al., 2009; Hsieh et al., 2013; Fenn et al., 2014; Morganti et al., 2016; Masuda et al., 2019). This functional heterogeneity is modulated by disease etiology, injury location, and the time elapsed since the original insult.

Based on these findings, the current consensus posits that microglia and macrophages in SCI exist on a spectrum as they respond to a lesion, with a balance of pro-inflammatory and anti-inflammatory reactivity being crucial to favorable SCI outcomes (Ransohoff, 2016; Brennan and Popovich, 2018). Deviations from this balance result in non-resolving pathologies, such as the glial scar that typifies SCI in mammals. Consequently, eminent researchers in the field of microglial biology recommend avoiding the M1/M2 terminology to prevent misinterpretation of data (Ransohoff, 2016; Paolicelli et al., 2022).

### 4.2. Microglial and macrophage responses to SCI

Following SCI, DAMPs and pro-inflammatory cytokines, such as ATP, IL-33, IL-1β, and TNF-α, trigger microglial reactivity and the adoption of a pro-inflammatory phenotype within the injured CNS microenvironment (Davalos et al., 2005; Rice et al., 2007; Kristina et al., 2009; Orr et al., 2009; Gadani Sachin et al., 2015). These reactive microglia migrate to the lesion site, undergo hypertrophy, and retract their ramifications, becoming morphologically indistinguishable from MDMs (Orr et al., 2009; Boche et al., 2013).

During the acute phase of SCI, reactive microglia release pro-inflammatory cytokines and chemokines, which augment astrocyte reactivity and recruit circulating neutrophils to the lesion site, exacerbating neuroinflammation and neuronal loss (Pineau and Lacroix, 2007; David et al., 2012; Kobayakawa et al., 2019; Pelisch et al., 2020). The subacute phase marks the beginning of MDM infiltration into the lesion site (Tran et al., 2018b). It is essential to delineate the distinct functions of microglia and MDMs in SCI. While MDMs are found within the lesion core, microglia localize along the margins of the fibrotic scar interfacing with the astrocyte border (Zhou et al., 2014). Microglia execute essential phagocytic and cytokine-producing functions while ensuring wound compaction in the fibrotic core and proper astrocyte scar formation, thereby limiting damage spread (Hines et al., 2009; Brennan et al., 2022). Conversely, MDMs mainly phagocytose debris and produce the cytokines and chemokines dictated by their polarization state without contributing to damage containment (David and Kroner, 2011). Only MDMs establish destructive physical contact with axons, inducing axonal dieback (Sarah et al., 2011; Evans et al., 2014). Furthermore, microglia repress genes in MDMs associated with ECM processing; in the absence of microglia, MDMs enhance ECM degradation and increase neuroinflammation (Brennan et al., 2022).

Phagocytosis is a prerequisite for wound healing post-SCI, mitigating neuroinflammation and promoting remyelination (Wang et al., 2015, 2022; David et al., 2018; Bellver-Landete et al., 2019; Lloyd and Miron, 2019; Fu et al., 2020). Microglia drive the early phagocytic response up to 3-dpi (i.e., until MDM infiltration starts), efficiently internalizing apoptotic and necrotic cell debris and myelin (Andrew and Samuel, 2014). By 7-dpi, MDMs at the lesion epicenter become the dominant phagocytic cell type, displaying superior phagocytic capabilities than microglia (Andrew and Samuel, 2014). Microglia also bolster the phagocytic functions of MDMs, whereas the latter actively suppress microglial phagocytosis and pro-inflammatory phenotypes (Greenhalgh et al., 2018). Preventing this macrophage-induced suppression of pro-inflammatory microglial polarization increases neuroinflammation and attenuates functional recovery (Greenhalgh et al., 2018). These findings indicate that the microglial-macrophage interplay operates to confine the lesion site, phagocytose and thereby eliminate pro-inflammatory toxic debris, and restore homeostasis.

However, MDMs process phagocytic debris less efficiently than microglia, leading to intracellular accumulation (Andrew and Samuel, 2014). Progressive myelin buildup inside MDMs is linked to their polarization toward pro-inflammatory states, akin to lipid-laden “foamy” macrophages observed in atherosclerotic plaques (Moore et al., 2013; Zhu et al., 2017; Milich et al., 2019). Longitudinally profiling MDM responses in SCI reveals a coexistence of pro- and anti-inflammatory populations in the subacute phase, whereas MDMs at 28-dpi exhibit a much stronger pro-inflammatory bias (Kigerl et al., 2009). These persistently activated macrophages within the fibrotic core are well-established contributors to the lack of wound resolution post-SCI (Wu et al., 2005; Li et al., 2022). The local SCI microenvironment drives these microglial and MDM phenotypes, thereby controlling disease outcomes. For instance, transitioning to an anti-inflammatory/pro-repair phenotype requires a shift in astrocytic signals from pro-inflammatory (TNF-α and IL-6) to anti-inflammatory (TGF-β and IL-4) (Norden et al., 2015). However, insufficient anti-inflammatory cytokines like IL-4 in the SCI microenvironment favor inflammation (Francos-Quijorna et al., 2016). Besides extracellular factors, phagocytosis of myelin debris promotes anti-inflammatory/pro-repair phenotypes, but TNF-α overrides this effect to sustain pro-inflammatory/anti-repair polarization (Kroner et al., 2014). Additionally, iron loading from RBC phagocytosis reverses the anti-inflammatory/pro-repair phenotype and increases TNF-α and inducible nitric oxide synthase (iNOS) levels, favoring inflammation (Kroner et al., 2014). Hence, therapies that appropriately modulate microglia and macrophage to achieve a balance between pro-inflammatory/anti-inflammatory polarization are needed (Shechter et al., 2009; Gensel and Zhang, 2015). Experimentally skewing microglia and macrophage polarization toward anti-inflammatory—by directly modulating their gene expression, utilizing stem cell transplantation, or manipulating the SCI microenvironment—has been shown to reduce axonal dieback, enhance angiogenesis, and improve functional outcomes after SCI (Busch et al., 2009, 2011; Francos-Quijorna et al., 2016; Pelisch et al., 2020; Gu et al., 2023; Ju et al., 2023).

Studies have also attempted to elucidate the intrinsic molecular pathways determining where microglia exist on their reactivity spectrum. Histone deacetylase 3 (HDAC3) is a key epigenetic regulator of microglial activation after SCI and skews their gene expression signature toward inflammation (Kuboyama et al., 2017; Huang D. et al., 2022). HDAC3 inhibition suppresses microglial pro-inflammatory cytokine secretion (Xia et al., 2017) and alleviates various CNS diseases, including SCI (Chen et al., 2018; Liao et al., 2020; Matheson et al., 2020; Bian et al., 2021; Zhao Y. et al., 2022; Lu et al., 2023). Microglia-specific HDAC3 knockout or administration of HDAC3 inhibitor RGFP966 exert neuroprotective effects in severe contusive SCI mouse models and increase the density of regenerating axons in the fibrotic scar 10-dpi (Kuboyama et al., 2017). Therefore, HDAC3 may constitute a therapeutic target to suppress pro-inflammatory/anti-repair microglial subsets.

### 4.3. Wound compaction by microglia

Hines et al. (2009) initially reported that microglia represent a frontline defense in the CNS, exhibiting rapid mobilization in response to injury to mitigate damage propagation. Depleting microglia via plexxikon molecules (PLX3397 and PLX5622) expands the SCI lesion size, disorganizes the astrocyte scar, and results in the spillover of ectopic clusters of MDMs into the surrounding white matter (Fu et al., 2020; Brennan et al., 2022), their morphology resembling “foamy” macrophages which are known to exert pro-inflammatory and neurotoxic effects (Wang et al., 2015; Zhu et al., 2017). Microglia-depleted mice also display worse locomotor recovery post-SCI, whereas stimulating microglia repopulation enhances recovery (Bellver-Landete et al., 2019; Brennan et al., 2022).

Microglia are thus emerging as pivotal orchestrators of the pro-homeostatic response following SCI. Numerous stereotypical functions of reactive astrocytes, including proliferation, cell adhesion, cytoskeletal reorganization, and inflammation, which are essential elements for proper astrocyte scar formation, are regulated by microglia, as evidenced by scRNA-Seq (Brennan et al., 2022). Mechanistically, reactive microglia in SCI physically contact scar-forming astrocytes to ensure proper glial scar formation and secrete IGF-1, which stimulates the proliferation of scar-forming astrocytes (Bellver-Landete et al., 2019). Microglial depletion by PLX5622 decreases astrocyte and OPC proliferation, resulting in a malaligned glial scar (Brennan et al., 2022).

Research from the Zhou Laboratory highlighted that microglia allow wound compaction function following through by their surface plexin-B2 receptor (Zhou X. et al., 2020). Upregulation of plexin-B2 contributes to the clear spatial segregation between the central fibrotic scar and astroglial border, whereas plexin-B2 deletion results in the intermingling of astrocytes and microglia at the lesion center and spillover of inflammatory components into the adjacent CNS tissue (Zhou X. et al., 2020).

### 4.4. Microglia and axonal regeneration

Transplanting microglia into mouse models of SCI has been shown to promote tissue preservation and enhance functional outcomes (Kou et al., 2018; Kobashi et al., 2020; Xia et al., 2022). However, many of these studies infer regeneration or remyelination from functional recovery rather than direct observation, while different mechanisms, such as synaptic remodeling, axonal sprouting, or regeneration through the lesion core, can underpin recovery.

Earlier in the discussion, we described how milder astrocyte manipulations that do not deplete astrocytes or completely abrogate their proliferation could enhance axonal growth. Since recent data show that microglia are crucial for forming a dense astrocytic scar, perhaps attenuating microglial functions can also “loosen” the astrocyte scar to allow for axonal regrowth. The study by Li Y. et al. (2020) demonstrated that SCI in neonatal mice results in the upregulation of GFAP-positive but loosely packed astrocytes with little evidence of hypertrophy and scar formation, in stark contrast to the compact astroglial border that forms in adult mice. This axonal regrowth-favoring glial scar was critically dependent on immature microglia, as depleting microglia in neonatal mice resulted in stronger astrocyte hypertrophy that was more compactly arranged, resulting in axonal regrowth failure and halted growth cones seen abutting astrocytes (Li Y. et al., 2020). Mechanistically, immature microglia release serine and cathepsin protease inhibitors, which reduced the deposition of astrocyte scar-inducing type I collagen and growth-inhibitory CSPGs (Li Y. et al., 2020). However, exogenously supplying protease inhibitors when transplanting mature microglia into the adult spinal cord improved axonal regeneration and functional recovery, but not to the extent seen after transplanting immature microglia, indicating that immature microglia also exert other currently unknown functions that promote scarless wound healing (Li X. et al., 2020).

It is important to mention that skewing the microglia/macrophage population toward anti-inflammatory/pro-repair phenotypes may not be enough to render the astrocyte border more conducive to axonal regeneration. For example, inhibiting HDAC3 by RGFP966 during the acute phase of SCI (0–2 dpi) significantly ameliorates neuroinflammation and enhances axonal sparing but does not affect GFAP expression levels, suggesting that the ability astrocytes to form a rigid, growth-blocking scar was unaltered (Kuboyama et al., 2017).

### 4.5. Current limitations in microglial research

Microglial investigations have struggled with the absence of specific markers. Recent discoveries of novel microglia markers, such as Tmem119, SLC2A5, Sall1, P2ry12, and FCRLS, and reporter mice have improved this situation (Bennett et al., 2016; Konishi et al., 2017; Jordão et al., 2019; Kaiser and Feng, 2019; Zhao et al., 2019; Masuda et al., 2020; McKinsey et al., 2020; Ruan et al., 2020). Still, concerns remain regarding the specificity of these markers, as some may be downregulated in reactive microglia or expressed in BAMs (Young et al., 2021; Ruan and Elyaman, 2022). Microglia-specific reporter mice such as Cx3cr1-Cre may also suffer from a lack of specificity by inadvertently labeling macrophages and glial cells (Zhao et al., 2019).

Loss-of-function experiments typically administer plexxikon CSF1R inhibitors, such as PLX3397 and PLX5622, that cross the BBB to deplete microglia (Elmore et al., 2014; Najafi et al., 2018; Green et al., 2020). However, PLX5622 depletes microglia and BAMs, hindering assessments of their differential contributions to CNS diseases (Montilla et al., 2023).

Thirdly, microglial depletion strategies in SCI animal models have yielded beneficial and detrimental effects on scarring, axonal regeneration, and functional recovery (Deng et al., 2022). Microglia primarily exert their beneficial functions within the first week of SCI, while activation beyond this phase proves harmful (Zhou X. et al., 2020). Therefore, divergent outcomes may arise from different timings of microglial depletion. The severity of manipulation is also important to consider: severe manipulations that either deplete microglia or their key pro-inflammatory and wound compaction functions in the acute and subacute phase of SCI are likely to be deleterious, whereas milder, timed manipulations in the chronic phase are more likely to be beneficial.

Finally, despite technological advancements enabling the examination of microglial heterogeneity at the single-cell level, the upstream regulators and functional consequences of this diversity remain unclear. Future research must also consider post-transcriptional and translational regulation of key transcripts in microglia, as a recent study demonstrated stringent post-transcriptional and translational control over pro-inflammatory gene transcripts (Boutej et al., 2017), such that solely considering transcriptomics may not accurately reflect the true nature of the microglial proteome. Based on this, Paolicelli et al. (2022) recommend adopting a multidimensional view of microglial biology that incorporates their epigenetic, transcriptomic, proteomic, metabolomic, and morphological states.

## 5. Fibroblasts

Unlike microglia and astrocytes, the roles of fibroblasts in SCI are only beginning to be elucidated. Fibroblasts in the adult CNS populate various spatial domains, including the meninges, choroid plexus, and perivascular spaces (Soderblom et al., 2013). Pericytes and endothelial cells also differentiate into fibroblast-like cells, contributing to fibrotic scar formation after CNS injury (Göritz et al., 2011; Zhou et al., 2019; Dias et al., 2021).

### 5.1. Fibroblast response to SCI

These distinct stromal cell populations are differentially recruited to the SCI lesion site, where they respond to fibrogenic factors such as TGF-β and elaborate ECM components, including collagen, laminins, fibronectin, and CSPGs leading to the formation of the fibrotic scar (Leask and Abraham, 2004; Klapka and Müller, 2006; Wynn, 2008; Ankeny and Popovich, 2009; Fawcett et al., 2012; Kawano et al., 2012; Soderblom et al., 2013; Gensel and Zhang, 2015; Wang et al., 2018).

This initial response seals off the injury site and limits CNS damage (Dias et al., 2018). Stromal cells are the primary producers of type I collagen in SCI, responsible for astrocyte scar formation (Hara et al., 2017). Moreover, interactions between EphB2 on astrocytes with ephrin-B2 on fibroblasts are believed to underpin the clear spatial segregation between the centrally located fibroblasts and astrocytes at the scar border (Bundesen et al., 2003). However, these interactions appear redundant since the astrocyte border persists even when fibrotic scar formation is attenuated; other cell types, such as microglia, are also involved in ensuring proper glial scar formation (Bellver-Landete et al., 2019; Dorrier et al., 2021).

Studies have shown that inhibiting key fibroblast functions after SCI enhances remyelination, axonal regeneration, and functional recovery (Pasterkamp et al., 1999, 2001; Hermanns et al., 2001; De Winter et al., 2002; Hellal et al., 2011; Cregg et al., 2014; Dias et al., 2018). Fibroblast-derived type I collagen induces the formation of the dense astrocytic scar, which chronically impedes axonal regeneration. Moreover, EphB2/ephrin-B2 interactions between astrocytes and fibroblasts can foster dysfunctional astrocyte reactivity and decrease synaptic plasticity and axonal regeneration after SCI (Li et al., 2017; Wu et al., 2021). Activated fibroblasts are also known to augment innate and adaptive immunity to promote inflammation and thereby delay wound healing in peripheral tissues (Ayazi et al., 2022), but similar mechanistic insights remain investigational in SCI.

### 5.2. Fibroblast origin and heterogeneity

Studies using ScRNA-Seq have demonstrated that meningeal fibroblasts from the dura, arachnoid, and pia are transcriptionally distinct (DeSisto et al., 2020). Recent studies have also shown three transcriptionally distinct clusters of perivascular fibroblasts in the healthy CNS (Garcia et al., 2022; Winkler et al., 2022). Whether fibroblasts derived from different origins—the meninges, perivasculature, or choroid plexus—have differential contributions to the fibrotic scar, glial scar persistence or resolution, and axonal regeneration/repair are important questions for future studies. In this regard, a recent study demonstrated that three transcriptionally distinct clusters of stromal cells—which the authors termed fibroblasts—accumulate at different stages after SCI (Gong et al., 2023). Cluster one fibroblasts begin appearing in the fibrotic scar by 7 days dpi, peak at 14 days dpi, and stay consistently elevated in the chronic phase. These fibroblasts exhibited a pro-inflammatory transcriptomic signature (Gong et al., 2023). Cluster 2 fibroblasts—enriched in genes encoding proteins involved in angiogenesis, ECM organization, TGF-β-related signaling, and collagen processing—accumulate in the center of the lesion at 3 days dpi (Gong et al., 2023). This study was the first to demonstrate that transcriptionally distinct subsets of stromal cells accumulate at different timepoints through the course of SCI. However, it is essential to note that, as already stated, documenting transcriptional heterogeneity does not necessarily imply differential functional contributions, which is an area that still requires further work.

The lack of specific markers to distinguish between different fibroblast lineages and perivascular cells such as pericytes and vascular smooth muscle cells (vSMCs) has hindered studies from delineating the distinct roles of each cell type in SCI. Studies on Glast-CreER mice—reporter mice that allow the inducible depletion of Glast1+ cells—ascribed a neuroprotective role to Glast1+ stromal cells in SCI (Göritz et al., 2011; Dias et al., 2021). The authors labeled these Glast1+ cells as pericytes, but astrocytes and fibroblasts also express Glast1 (Regan et al., 2007; Vanlandewijck et al., 2018). Col1a1-GFP transgenic mice demonstrate that stromal cells within the fibrotic scar are derived from the meninges rather than the perivasculature and do not express pericyte markers such as NG2 (Soderblom et al., 2013), supporting the idea that the studies describing Glast1+ pericyte functions may have been studying fibroblasts. The Gong et al. (2023) study discussed above also utilized Glast1 positivity to define stromal cell identify, and hence we caution against considering these cells as fibroblasts or pericytes until further results prove otherwise.

Similarly, although NG2^+^ perivascular cells are known to perform essential functions in angiogenesis and fibrotic scar formation after SCI (Hesp et al., 2018), NG2 is expressed by pericytes, perivascular fibroblasts, vSMCs, and OPCs (Bergers and Song, 2005). Therefore, tracing stromal cell lineage based solely on Glast1 or NG2 expression lacks specificity, and the origin of stromal cells in the fibrotic scar remains debatable. Dorrier et al. (2021) utilized cell lineage-tracing technologies to demonstrate that perivascular fibroblasts—not pericytes or vSMCs—contributed to fibrotic scar development in EAE mouse models. It would be valuable to apply similar methodologies to SCI to unequivocally discern the origin of stromal cells in the fibrotic scar.

## 6. Oligodendrocyte progenitor cells

Oligodendrocyte loss ensues immediately after SCI, and their apoptosis continues into the subacute and chronic phases in various animal models (Li et al., 1999; Almad et al., 2011; Pukos et al., 2019). Demyelination of spared axons is thus a prevalent feature post-SCI and contributes to neuronal impairment by compromising axonal conduction even in anatomically incomplete lesions (Pukos et al., 2019). The mechanistic underpinnings of this phenomenon and how spared axonal function can be restored have attracted much research interest, especially given the fact that maintaining the functional integrity of a few axons could significantly better neuronal function (Schucht et al., 2002; Kakulas, 2004). Hence, promoting remyelination, in which OPCs are crucial, has long been sought after as a potential therapeutic strategy. NG2^+^ OPCs cells are spread throughout the CNS (Nishiyama et al., 1999, 2016), actively interact with neurons (Bergles et al., 2000; Sahel et al., 2015), and sustain oligodendrocyte turnover and remyelination (Watanabe et al., 2002).

### 6.1. OPC response to SCI

Oligodendrocyte progenitor cells acutely mount a robust proliferative response that peaks at 5 dpi, accumulating in the lesion penumbra alongside astrocytes (McTigue et al., 2001; Zai and Wrathall, 2005; Barnabé-Heider et al., 2010; Hesp et al., 2015). Ependymal cells, the NSCs of the spinal cord, also give rise to OPCs in SCI (Meletis et al., 2008). Multiple factors in the SCI microenvironment, such as TNF-α and WNTs, drive OPC proliferation (Tripathi and McTigue, 2007; Moore et al., 2011; Miron et al., 2013; Burda and Sofroniew, 2014; Hackett et al., 2016; Miron, 2017) but concomitantly impair their differentiation into mature oligodendrocytes as a result of enhanced β-catenin signaling (Rhodes et al., 2006; Liu et al., 2008; Hill et al., 2013). Moreover, OPCs express RPTPσ, which can bind CSPGs to inhibit OPC differentiation into oligodendrocytes (Ranjan and Hudson, 1996; Siebert and Osterhout, 2011; Pendleton et al., 2013; Karus et al., 2016).

Oligodendrocyte progenitor cells also differentiate into remyelinating Schwann cells, although it should be stated that the impact of this process on functional recovery remains controversial and may not be significant (Duncan et al., 2018, 2020). Furthermore, OPCs have been shown to differentiate into astrocytes after SCI (Suzuki et al., 2017; Duncan et al., 2020), which express anti-inflammatory and anti-apoptotic proteins such as crystallin alpha B (Hou et al., 2022), indicating that OPC-derived astrocytes may be neuroprotective. Fate-mapping NG2^+^ cells reveal that 25% of OPCs differentiate into astrocytes in a contusive SCI model (Hackett et al., 2018). In contrast, this trajectory is less likely in a stab or transection SCI model or EAE (Hackett et al., 2018), indicating that the disease-specific microenvironment is crucial in determining OPCs fate.

Dorrier et al. ablated proliferating fibroblasts in the fibrotic scar using a transgenic herpes simplex virus thymidine kinase combined with ganciclovir (HSV-TK/GCV) EAE mouse model. After fibroblast ablation, there was a significant increase in the infiltration of Olig2-positive OPCs into the inflamed lesion (Dorrier et al., 2021). These results were corroborated by *in vitro* findings that collagen-producing fibroblasts significantly reduce the migration of OPCs across a transwell insert (Dorrier et al., 2021). Therefore, the dense stromal cell and ECM presence in the SCI scar may limit OPCs to the lesion periphery, restricting their access to demyelinating axons.

### 6.2. OPCs beyond remyelination

Running contrary to the remyelinating response of OPCs, NG2 is an inhibitor of axonal regeneration (Dou and Levine, 1994; Petrosyan et al., 2013). NG2^+^ cells have been visualized besides dystrophic axon end-bulbs, which can be reversed by administering an anti-NG2 antibody (McTigue et al., 2006; Tan et al., 2006; Filous et al., 2014). However, NG2^+^ cells also reduce macrophage-induced axonal dieback (Busch et al., 2010), possibly by forming synapse-like connections with the tips of transected axons (Fünfschilling et al., 2012; Angela et al., 2014). NG2^+^ cells also produce ECM components such as fibronectin and laminin that protect axon growth cones from the neuroinflammatory milieu (Angela et al., 2014; Tran et al., 2018b). However, this acute neuroprotection appears to be at the expense of long-term regeneration, as NG2^+^ cells and the ECM chronically entrap axon growth-cones to hamper regeneration (Busch et al., 2010; Di Maio et al., 2011; Bradke et al., 2012; Son, 2015; Hackett and Lee, 2016). Entrapped axon growth cones have persisted as long as 40 years post-SCI (Silver and Miller, 2004; Tom et al., 2004), indicating that this entrapment is permanent. Freeing trapped dystrophic axon growth cones may constitute a therapeutic approach to enhance regeneration (Tran et al., 2018b).

Oligodendrocyte progenitor cells become reactive in the SCI microenvironment and secrete MMP-9, increasing BSCB permeability and enhancing neuroinflammation (Seo et al., 2013). Reactive OPCs may also be sources of pro-inflammatory cytokines and chemokines that induce pro-inflammatory reactive states in microglia and macrophages (Kucharova and Stallcup, 2015). The spatial localization of OPCs also provides insight into their intercellular communication: OPCs reside in the scar border alongside proliferating astrocytes, which interface with reactive microglia at the margins of the fibrotic scar (Keirstead et al., 1998). Ablating NG2^+^ glia in the lesion penumbra significantly reduces astrocyte hypertrophy and GFAP expression, resulting in disorganization of the glial scar, expansion of the lesion site, and worse neurologic outcomes (Hesp et al., 2018). These results align with transgenic experiments showing that drastic attenuation of each cell type is detrimental to the host injury response.

Milder manipulations of OPCs without their depletion reveal more specific functions of OPCs. A recent study showed that OPC-specific β-catenin deletion in tamoxifen-inducible cre-recombinase mice enhances OPC differentiation into mature oligodendrocytes and significantly reduces astrocyte hypertrophy and GFAP expression, fostering a growth-permissive microenvironment that promotes axonal regeneration and improves recovery of hindlimb motor function after SCI (Rodriguez et al., 2014). β-Catenin deletion in OPCs also polarized microglia and macrophages to anti-inflammatory/pro-repair phenotypes (Rodriguez et al., 2014). Moreover, by injecting adeno-associated virus (AAV) containing Wnt3a+GFP or Wnt5a+GFP into uninjured spinal cords of female C57BL/6J mice, the authors showed that Wnt3a-induced β-catenin signaling significantly increased the infiltration and proliferation of OPCs around the injection site (Rodriguez et al., 2014). The microglia infiltration also substantially increased around the injection site, while the density of GFAP^+^ astrocytes remained unaltered (Rodriguez et al., 2014).

These results suggest that OPCs are part orchestrators of the injury, particularly myeloid cell, response following SCI. Dampened microglial responses could also explain the decrease in GFAP+ astrocyte hypertrophy and density in β-catenin-KO mice, as these cells facilitate reactive astrocytosis at the lesion site (discussed above). The combination of a looser astrocyte border and significant reduction of CSPGs can account for improved axonal regeneration and density around the lesion site.

### 6.3. Current limitations

The lack of OPC-specific markers is a major hindrance in dissecting their contributions to SCI pathology and repair. NG2 and PDGFRα are commonly used OPC markers but are also expressed by stromal cells such as pericytes (Bergers and Song, 2005). For example, Hesp et al. (2018) utilized an HSV-TK/GCV transgenic model to deplete NG2^+^ stromal cells (pericytes or fibroblasts) in the fibrotic scar and NG2^+^ OPCs in the glial scar. Eliminating NG2^+^ cells in the lesion epicenter completely abolished the fibrotic scar, and loss of dividing NG2^+^ OPCs disrupted astrocytic scar formation. However, given that NG2^+^ stromal cells also ensure proper astrocyte border formation (Hesp et al., 2018), this approach could not delineate the differential effect of stromal cells and OPCs. Moreover, current genetic mouse lines, which allow inducible attenuation or enhancement of OPC-mediated remyelination, also affect glial scarring, astrocyte reactivity, and neuroinflammation (Duncan et al., 2020). Future studies utilizing combinatorial strategies of NG2/PDGFRα reporter mice and localization and imaging techniques supplemented with an array of cell surface markers will better identify NG2^+^ OPCs and allow their specific contribution to be dissected at higher resolution.

## 7. A neuro-centric view of axonal regeneration

Neurobiologists have long known the ability of axons in the developing CNS to grow far-reaching axons. Peripheral nervous system (PNS) axons also regenerate effectively and reach innervation targets after injury, whereas adult CNS neurons do not (Broude et al., 1997). Animals such as the CAST/Ei mouse strain display excellent axonal regeneration after injury, accompanied by specific changes in neuron gene expression programs not seen in control mice that exhibit poor regeneration (Omura et al., 2015). Therefore, studies have cited gene expression differences as essential factors in the poor regenerative capacity of the adult mammalian CNS. Multiple ground-breaking studies over the past decade have uncovered numerous vital regulators of axon growth programs in SCI at the transcriptional, translational, and epigenetic levels, including PTEN-mTOR, SOCS3-STAT3, cAMP, REST/NRSF, and many others (Qiu et al., 2002; Park et al., 2008; Liu et al., 2010; Sun et al., 2011; Cheng et al., 2022). Excellent reviews on the neuron-intrinsic regulators of axonal regeneration are referenced here (He and Jin, 2016; Mahar and Cavalli, 2018; Bradke, 2022; Zheng and Tuszynski, 2023).

The Sofroniew Laboratory observed that a combination of factors underpins CNS regeneration failure: insufficient growth factors, chemoattractive substrates, and a failure to active pro-regenerative gene signatures in neurons (Anderson et al., 2018). Supplying all three elements together—but not individually—significantly enhances axonal regeneration past the astroglial and fibrotic scars more than 140-fold greater than in control mice (Anderson et al., 2018). Therefore, delivering a variety of cells and neurotrophic factors, such as through biomaterial-based approaches (Liu S. et al., 2018; Courtine and Sofroniew, 2019; Guijarro-Belmar et al., 2022), is a promising avenue to promote axonal regeneration and functional recovery.

## 8. Concluding remarks and perspectives

This review focused on the dual roles of the SCI scar, an acutely beneficial and chronically pathological one. Indiscriminate targeting of the essential cellular components of the SCI scar is deleterious in animal models but does not discount the chronically detrimental roles of glial and stromal cells. Indeed, more specific and milder manipulations of cellular constituents of the SCI scar enhance axonal regeneration and functional recovery. Concomitantly, advancements in single-cell technologies have revealed profound cellular heterogeneity in the SCI scar, which underscores the importance of cell- and context-specific therapeutic manipulations. To develop such therapeutic strategies, it is essential to further characterize the regulation and functional significance of cellular heterogeneity.

One can classify the different therapeutic strategies that may be employed to target the host response to SCI: (1) enhancing neuron regenerating capacity; (2) targeting extracellular regeneration inhibitors such as CSPGs and myelin; (3) targeting the glial cell responses (particularly astrocytes and microglia); and (4) targeting the central fibrotic core. Unlike the first three, the fibrotic core has received relatively little attention regarding its therapeutic value. The origin of stromal cells, their apparent heterogeneity at the lesion site, and the effects of milder manipulations (rather than transgenic ablation) of stromal cells are poorly understood. Exploring these aspects may pave the way for future therapies employing combinatorial approaches harnessing the innate regenerative capabilities of neurons and promoting a pro-repair microenvironment within the injured spinal cord.

We would also like to see the impact of biological aging hallmarks such as cellular senescence be further fleshed out in explaining divergent SCI responses across lifespan. Transgenic models such as INK-ATTAC and p16-3MR mice are available that allow the specific tracking and inducible depletion of senescent cells (Baker et al., 2011; Demaria et al., 2014) and would be helpful in determining the particular cell types undergoing senescence in SCI and the effects of their depletion in different SCI phases. Given the rapid evolution of senolytics from benchwork into clinical trials, we feel it prudent to investigate the role of cellular senescence in SCI to unveil potentially another therapeutic strategy improve SCI outcomes.

Ultimately, only rigorous testing will uncover novel and potentially groundbreaking therapeutic targets, revolutionizing our ability to enhance regeneration and improve outcomes for humans affected by spinal cord injuries.

## Author contributions

AS conceptualized the manuscript. AS, IA, HM, and TS prepared the initial draft and designed the figures. AS, KA, and AY reviewed the manuscript and prepared the final version. All authors read and approved the final version of this manuscript.
